# Optimising Weight Management Advice for Adults Prescribed Antidepressants: A Qualitative Interview Study of Experiences

**DOI:** 10.1111/inm.70068

**Published:** 2025-05-28

**Authors:** Angela Rodrigues, Nikita Kohli, Charlotte Watson, Katie Boynton

**Affiliations:** ^1^ Department of Psychology Northumbria University Newcastle upon Tyne UK; ^2^ Newcastle Upon Tyne Hospitals NHS Foundation Trust Newcastle upon Tyne UK

**Keywords:** adverse effects, antidepressant use, mental health, qualitative research, weight gain

## Abstract

Antidepressants are widely prescribed for depression and anxiety, yet common side effects like weight gain can adversely affect physical and psychological health, highlighting the need for tailored weight management strategies for users. This study aims to explore these experiences, identify barriers and facilitators to effective weight management, assess the availability of weight management services, and understand the role of healthcare professionals in providing support. We conducted semi‐structured interviews in the United Kingdom between May–August 2022 (female participants) and December 2023–February 2024 (male participants) with 13 adults prescribed antidepressants aged 19–62. Participants were purposively sampled for variation according to age, gender, type of medication, length of prescription and weight gain/loss. We audio recorded each interview, transcribed it verbatim, and analysed transcripts using thematic analysis. Participants' experiences emphasised the significant role of healthcare professionals in supporting weight management, with impacts ranging from helpful to counterproductive. Adults on antidepressants faced unique practical and psychological challenges, making weight management more complex than typical weight loss efforts. Many struggled to balance mental health needs with concerns about weight gain as a side effect, highlighting a strong need for more comprehensive and proactive support from healthcare providers in addressing medication‐related weight changes. This study identifies key areas for supporting weight management in antidepressant users: enhancing proactive healthcare support, tailoring strategies to the unique challenges of antidepressant use and balancing mental and physical health priorities. Early intervention and regular monitoring could improve weight management experiences, highlighting the need for targeted interventions and a proactive role for healthcare providers.

## 1 Introduction

Antidepressants are widely prescribed for conditions such as depression and anxiety (Kirsch et al. 2008; Strawn et al. 2018; Kennedy et al. 2016). Recent studies have highlighted an increase in antidepressant prescriptions globally (Brauer et al. 2021) and, as of 2022, the highest rates of antidepressant consumption were observed in Iceland, Portugal, and the United Kingdom, with the United Kingdom reporting approximately 138 defined daily doses per 1000 people (OECD 2019). Despite being an effective treatment for various mental conditions, one common side effect of antidepressants is weight gain (Chevance et al. 2022), which can lead to metabolic disturbances (Carvalho et al. 2016) and negatively impact both physical health and psychological well‐being (Read et al. 2014).

## 2 Background

Antidepressants are widely prescribed for conditions such as depression and anxiety (Kirsch et al. 2008; Strawn et al. 2018; Kennedy et al. 2016). Recent studies have highlighted an increase in antidepressant prescriptions globally (Brauer et al. 2021) and, as of 2022, the highest rates of antidepressant consumption were observed in Iceland, Portugal, and the United Kingdom, with the United Kingdom reporting approximately 138 defined daily doses per 1000 people (OECD 2019). Despite being an effective treatment for various mental conditions, one common side effect of antidepressants is weight gain (Chevance et al. 2022), which can lead to metabolic disturbances (Carvalho et al. 2016) and negatively impact both physical health and psychological wellbeing (Read et al. 2014).

In the United Kingdom, mental health care has evolved significantly, shifting from inpatient‐reliant services to a more community‐based approach (NHS England 2019). In the United Kingdom, the National Health Service (NHS) Long Term Plan has committed to increasing mental health funding, improving access to psychological therapies, and integrating mental and physical health services (NHS England 2019). With 9 out of 10 adults with mental health conditions being supported in primary care (NHS England 2019), antidepressants remain a key component of treatment. However, despite the growing recognition of the link between antidepressant use and weight gain, there is limited structured support for individuals managing this side effect within routine mental health care.

Previous qualitative studies have explored patients' experiences with antidepressants, focusing on decisions to start medication (Maxwell 2005), effects on users' ‘sense of self’ and long‐term use (Knudsen et al. 2002). Gibson et al. (2016) revealed diverse experiences among antidepressant users, ranging from life‐saving to issues with side effects, highlighting the need for personalised mental health care. Another study (Schofield et al. 2011) explored factors influencing patients' decisions about taking antidepressants, revealing that initial use often followed a crisis, with patients feeling they had no choice. Through trial and error, patients became more knowledgeable about their condition and treatment, eventually making more informed decisions about medication use.

Recent research has investigated the impact of behavioural weight management programs to combat weight gain or to support weight loss in patients taking psychiatric medications (Wharton et al. 2019; Imayama et al. 2013). One study found that participants in a weight management program lost substantial weight regardless of antidepressant or antipsychotic use (Wharton et al. 2019). Similarly, another study demonstrated that antidepressant use did not hinder adherence or improvements in weight, body measures, or metabolic markers within a structured diet and exercise program for postmenopausal antidepressant users (Imayama et al. 2013). Together, these findings underscore the potential for effective weight management when antidepressants or antipsychotics are involved.

Recent studies have highlighted the need for personalised mental health care that addresses both the mental and physical health concerns of patients (Gibson et al. 2016; Schofield et al. 2011). However, despite growing awareness of the impact of antidepressant‐induced weight gain, limited research has been conducted to understand patients' lived experiences and the specific strategies they employ for managing weight within this context. This research aims to address this gap and provide details on how individuals cope with and manage weight gain while on antidepressants, especially given the rising number of prescriptions. These details will be helpful to inform clinical practice by providing an understanding of how antidepressant use impacts weight management and to guide healthcare professionals in supporting patients with more patient‐centred weight management strategies. This research will be particularly relevant to healthcare providers, mental health professionals, policymakers and researchers working in the fields of mental health and obesity management. It will also provide relevant information for individuals who are prescribed antidepressants and who may be struggling with weight gain, as it can inform future strategies to improve patient care and reduce barriers to treatment adherence. By exploring these experiences, this study aims to identify barriers and facilitators to effective weight management, assess the availability of weight management services and understand the role of healthcare professionals in providing support. This research seeks to support improvements in weight management strategies for antidepressant users, contributing to the development of more effective, personalised approaches.

## 3 Methods

### 3.1 Study Design

Ethical approval for this study was granted from Northumbria University Faculty of Health and Life Sciences. The study utilised semi‐structured, one‐to‐one online interviews to explore participants' experiences of weight management while using antidepressants. This method was chosen to allow for in‐depth exploration of individual experiences, acknowledging that each participant's journey with weight management and antidepressant usage is unique. This research adopted a qualitative approach underpinned by critical realism. The critical realist stance recognises an external reality while acknowledging the subjective meanings and discourses present in participants' experiences (Fletcher 2017). Given the interpretive nature of qualitative research, we also acknowledge the positionality of the research team. Some researchers involved in data collection had personal experiences with antidepressants and weight changes, positioning them as partial insiders. This dual perspective may have facilitated rapport with participants while also requiring reflexivity to minimise potential biases in data interpretation. The study is reported in line with the Standards for Reporting Qualitative Research (SRQR) checklist from the EQUATOR Network website.

### 3.2 Participants

Participants were recruited through convenience sampling via social media platforms, local charities' social media, and posters around Newcastle upon Tyne, UK. All advertisements included a QR code and link to a screening survey. This survey provided study information, collected demographic data and obtained initial consent. Eligible participants (18+ years, self‐declared currently taking antidepressant medication, and living in the United Kingdom) who provided contact information were invited via email to schedule interviews. Weight changes (gain/loss) were self‐reported. Informed consent was reconfirmed verbally before interviews.

In total, 37 individuals expressed interest, and 13 agreed to participate in interviews. Respondents included seven females (aged 18–64) and six males (aged 19–62). Of those who did not participate, reasons included time constraints, not meeting inclusion criteria and non‐response to interview invitations. A higher rate of no‐shows was noted among male participants.

Interviews were conducted remotely via Zoom and MS Teams and carried out by the researchers K.B. (Female, Health Psychology MSc, between May and August 2022, focusing on female participants) and C.W. (Female, Psychology BSc, between December 2023 and February 2024, focusing on male participants).

### 3.3 Data Collection

One‐to‐one, online semi‐structured interviews were conducted by two female researchers (K.B. and C.W.). The researchers were not previously known to participants. The interview process was thoughtfully designed to create a comfortable environment, encouraging participants to openly share their weight management experiences with an emphasis on improving services. The interview guide (Supporting Information) aimed to explore topics such as history and effects of weight changes, beliefs about medication and weight, experiences with healthcare professionals, barriers, challenges and preferences, and was informed by previous work in this area (Kemp et al. 2025). The research process, including the development and refinement of the topic guide through pilot testing, was supervised by an experienced qualitative researcher (A.R.) to ensure methodological rigour. Participants consented to audio recording and transcription. Interviews lasted an average of 40 min (range 17–55 min). Recordings were deleted after transcription, and transcripts were reviewed for accuracy and anonymised before analysis.

### 3.4 Data Analysis

Data analysis was conducted manually using Microsoft Word for coding. Data analysis was conducted using reflexive thematic analysis (Braun and Clarke 2019). To ensure a rigorous analysis of the data, a structured six‐phase approach was applied: (1) Familiarisation with the data involved repeatedly reading the transcriptions to gain a deep understanding. (2) Two authors (N.K.: Health Psychology MSc; A.R.) then generated initial codes through an inductive process, creating descriptive codes and staying close to the participants' original language about their experiences of weight management advice. If codes were linked to more than one area, they were categorised under the most relevant theme via discussion throughout the analysis process (N.K. and A.R.). (3) Similar codes were clustered together, refining where necessary to address duplication and improve clarity (N.K.). (4) These themes, along with coded transcripts and illustrative quotes, were shared with all co‐authors for review and refinement. (5) The research team discussed and agreed on theme definitions and names. (6) Finally, the report was produced, with themes refined and clarified iteratively to ensure the results were clearly communicated in terms of factors influencing participant's' experiences of weight management advice.

## 4 Results

### 4.1 Participants and Descriptive Data

Thirteen participants took part in the study, consisting of six males and seven females. The duration of antidepressant use varied significantly, ranging from less than 1 year to over 5 years. Participants were taking a variety of antidepressant medications, including selective serotonin reuptake inhibitors (SSRIs) such as sertraline, citalopram and fluoxetine, as well as a serotonin‐norepinephrine reuptake inhibitor (SNRI) like duloxetine. Some participants were taking other antidepressants, including lofepramine and mirtazapine.

Most participants reported weight gain, with the exception of four: three experienced no change in weight, and one reported weight loss (notably, this participant was an avid gym user who received supportive healthcare guidance). Participant ages ranged from 19 to 62 years, with a mean age of 29.3. Two participants chose not to disclose their age. Among the 13 participants, seven participants are long‐term users of antidepressants. It is noteworthy that nearly all the long‐term users experienced weight gain, whereas only three out of six participants who are short‐term users reported the same. There was also consideration of the potential relationship between weight gain and age. However, no relationship emerged, as both younger and older participants experienced weight gain. Table 1 provides a detailed overview of all the participants' demographics collected.

**TABLE 1:** Demographic characteristics of all participants.

Participant	Age	Gender	Medication	Length of prescription	Weight gain/loss
1	29	Male	Sertraline	> 1 year	Loss
2	26	Male	Sertraline	+5 years	Gain
3	19	Male	Citalopram	> 1 year	Gain
4	62	Male	Fluoxetine	+5 years	Gain
5	20	Male	Sertraline	1–2 years	No changes noted
6	20	Male	Sertraline	1–2 years	Gain
7	21	Female	Sertraline	3–4 years	Gain
8	—	Female	Citalopram	3–4 years	Gain
9	21	Female	Sertraline	3–4 years	No changes noted
10	40	Female	Duloxetine	< 1 year	Gain
11	21	Female	Citalopram	< 1 year	No changes noted
12	—	Female	Lofepramine and mirtazapine	3–4 years	Gain
13	43	Female	Sertraline	3–4 years	Gain

**FIGURE 1:**
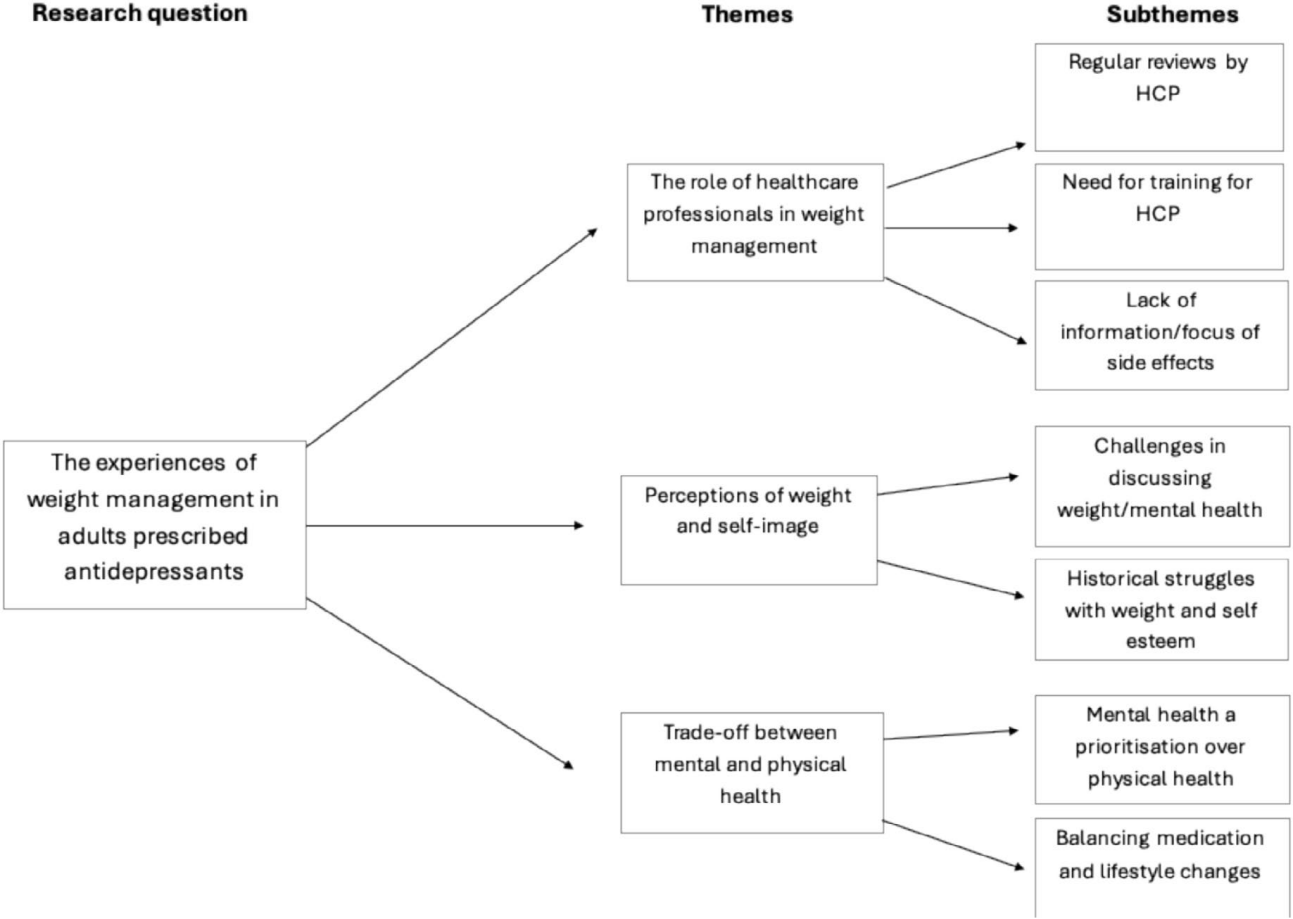
Diagram showing thematic map.

Inductive thematic analysis identified three core themes; these themes are: (1) The role of healthcare professionals in weight management, (2) perceptions of weight and self‐image and (3) trade‐off between mental and physical health. Figure 1 displays the thematic map, showing each theme and its sub‐themes.

#### 4.1.1 Theme 1: The Role of Healthcare Professions

This theme highlights participants' perspectives on the critical role that healthcare professionals play in helping treat the weight‐related effects of antidepressant drugs. Whether the influence was positive or negative, participants stated that their healthcare professionals significantly influenced how they managed these side effects. Three subthemes emerged from this theme, emphasising participants' desire for more support from their healthcare providers in managing their weight, recognising the significant role they play. Participants also expressed concerns about the lack of information regarding potential side effects and how this made them feel.

The first subtheme emerged from participants who emphasised the importance of having regular reviews and check‐ups with healthcare professionals. They expressed a clear need for these appointments to be initiated or conducted more frequently and more in depth.

I think when I was started taking it, you had monthly reviews. Uhm, and then after you've been on it for a while, it kind of changes to like yearly or like 6 months. And maybe those kind of review sessions could be used to monitor side effects, because I feel like those sessions are most sort tailored to ‘is a medication working’ good great. (Participant 9)

Participant 9 is expressing a concern that while initial monthly reviews after starting medication were helpful, the frequency of these reviews decreased over time. She suggests that these sessions, which currently seem to focus on whether the medication is effective, should also be used to address side effects. She feels that the current approach misses an opportunity to discuss and manage the side effects of the medication, which could be better integrated into these check‐ups.

Even just having a more regular kind of check‐up just being like. Because really, the kind of review was just like, how are you feeling? And that's it. It's just like if they could probe a bit more just being like are you experiencing any of the side effects that I'm going to listen right now, just be open and honest, and we're gonna look at that. (Participant 2)

Participant 2 experienced weight gain after commencing sertraline and emphasised the need for regular, more in‐depth reviews. He believes that healthcare professionals should make a greater effort to understand the participant's experiences, creating an environment where he feels comfortable being open and honest, ultimately allowing him to receive the necessary support.

While most participants expressed a need for more support and regular check‐ups, one participant's experience demonstrated the potential positive impact of receiving adequate healthcare guidance. This participant, an avid gym user, experienced weight loss rather than gain after receiving support from a healthcare professional following an initial negative interaction. This contrasting case highlights how regular, supportive healthcare guidance, combined with personal resources, can lead to different outcomes in managing antidepressant‐related weight changes.

‘I tend to just exercise it out and, in the end, I tend to lose more weight’. ‘My heal care profession actually was really very, very communicative … but he was really checking up on me and. Yeah, he was really interested in the side effects as well’. (Participant 1)

The second subtheme underscored the importance of training for healthcare professionals, particularly in communication. It highlighted the need for healthcare providers to receive training that enables them to engage with participants in a way that makes them feel supported.

Training thereby they could make easy communication and all that this be more professional in their job. I think I I I would love that because it's really helpful when it comes to weight management there. (Participant 1)

This participant, who experienced weight loss, highlights the potential for healthcare professionals to receive further training on communication skills to better support individuals managing their weight while on antidepressants. Their statement reflects a strong desire for effective, compassionate communication from their healthcare provider to help navigate the challenges associated with their weight changes and antidepressant use.

The final subtheme focused on the lack of information and attention given to the medication's side effects. Many participants felt there was a lack of emphasis on how significant weight gain/loss could be as a side effect. Some reported not being informed about the possibility of weight gain at all, while others were only given a leaflet with the information, with no further discussion from their healthcare provider. Some participants even expressed that had they been fully aware of the severity of weight gain as a side effect, they might have reconsidered accepting the antidepressants offered.

[not having had a conversation about side effects] Oh yeah, disappointed. Of course, yeah. Yeah, I mean, there should have been. I should have been made aware of that. I see on the leaflet it says about weight now but I didn't know. (Participant 4)

This participant expresses disappointment about not being informed that weight gain could be a potential side effect of the medication. They feel their healthcare provider should have explicitly mentioned this, as they were unaware of it at the time of prescription. This participant believes they should have been fully informed from the start.

#### 4.1.2 Theme 2: Perceptions of Weight and Self‐Image

This second theme reflects participants complex relationship between weight, self‐image and mental health. It captures a range of perspectives, including those who have gained, lost or maintained their weight while on medication, and how these experiences influence their sense of self. This theme also highlights the emotional responses tied to individuals' weight, particularly when told or experienced that the medication could have an impact on this.

One of the subthemes that emerged from this theme explores the difficulties participants faced when discussing their weight and mental health with a healthcare professional. Many participants expressed discomfort or hesitation in bringing up these topics, often due to personal feelings of shame, embarrassment or insecurity related to their self‐image.

(talking about communicating with HCP about weight) ‘Uncomfortable. And yeah. I wouldn't discuss that with them in regard to all that’. ‘To be honest though, I think that's why I don't discuss it because for me, rightly or wrongly, I don't know if. It's just my perception. I think they just sit there and think well, you eat too much or drink too much’. (Participant 7)

Participant 7 conveys a strong sense of discomfort when discussing their weight with their healthcare practitioner, admitting to avoiding the topic due to a fear of judgement. This reflects a broader issue of stigma, where this participant feels that their weight concerns are not fully acknowledged within the context of their mental health and medication use. Their response also suggests feelings of shame and self‐blame regarding their weight and how they are managing this.

The second subtheme emphasises participants' continued challenges with weight management, which are often linked to earlier experiences that have negatively impacted their self‐esteem. This also captures the frustration and distress felt by those who had hoped that antidepressants would improve their mental health, only to find that potential weight changes caused by the medication could instead have a further negative impact, potentially worsening their mental health.

Especially through school, were so intrinsically tied to the way I look, my body, especially so then to have this medication that's supposed to, not entirely fix it, but definitely make it better to then be faced with, you're gonna gain weight now, it's like especially like with body dysmorphia and stuff like that. It's just like it's one extra stumbling block to deal with. So, it was just kind of like, oh, really. (Participant 3)

This participant is expressing how their struggles with body image, particularly during school, were linked to their self‐esteem. Instead of providing relief, the medication introduces another obstacle, making it even harder to navigate their relationship with their body. Their reaction of ‘oh, really’ suggests frustration or disappointment, as they feel like they are facing yet another hurdle in their journey toward better mental health.

#### 4.1.3 Theme 3: Trade‐Off Between Mental and Physical Health

The final theme highlights the participants' internal conflict over the use of antidepressants, as they struggle with prioritising their mental health over their physical well‐being. Many participants recognised the importance of managing their mental health with medication but were concerned about the potential side effects, such as weight gain, which could negatively impact their physical health.

One of the subthemes highlighted how participants, at the time of their prescription, chose to prioritise their mental health over their physical health. Many felt their mental health had deteriorated to an extent that, despite potential side effects, they chose to take the medication in hopes of easing their struggles.

At that point I was just so desperate to get help, I didn't really care that much because I was in such a bad place, to the point where I wasn't leaving the house at all, and it was just really difficult to do anything. So, I was just like whatever I'll deal with it. Somehow, I just need to stop feeling this way first and then I'll deal with that. So, like I wasn't happy about it, but at that point I just didn't care enough not to take the medication. (Participant 11)

This participant explains that, at the time, they were in such a desperate mental state that concerns about weight gain as a side effect of medication became insignificant. It was extremely difficult for them to carry out routine tasks, such as leaving the house, because their mental health had deteriorated significantly. Consequently, they prioritised improving their mental health over any potential physical consequences. Participants were unhappy about the potential for weight gain, but they believed that this was a trade‐off they were prepared to make to prioritise their mental health.

The second subtheme explores the difficulties participants encountered in managing their weight while balancing antidepressant use and efforts to maintain a healthy lifestyle through diet and exercise.

It's OK, it's. I know, I know, you know, it's not only tablets. It's uh, if I was on healthy diet and if I was doing more exercise, of course I would lose weight, but I mentally wasn't well for years for quite few years and making myself think of healthy habits and exercise when I'm struggling with other to myself, more important things. My weight and a healthy eating plan is the last thing on my mind. (Participant 8)

Some participants experienced weight gain while taking antidepressants and acknowledges that a healthy diet and exercise are important for weight management. However, they explain that their mental health struggles have made it challenging to prioritise these aspects. They note that focusing on healthy lifestyle changes has become secondary to addressing their mental health, making it difficult to find a balance. This highlights the challenge many participants faced in balancing the effects of antidepressants with the pursuit of healthier lifestyle choices to manage both their mental and physical health.

## 5 Discussion

### 5.1 Summary of Findings

The findings of this study offer valuable understanding into how individuals prescribed antidepressants navigate weight management, particularly highlighting the crucial role of healthcare professional support and systemic barriers in their experiences. Participants' experiences demonstrated the significant influence healthcare professionals have on their ability to manage weight, with this impact varying between positive and negative outcomes. Adults taking antidepressants interviewed faced a complex array of practical and psychological challenges, indicating that weight management in this context is more complicated than general weight loss efforts. Participants experienced a notable dilemma in balancing their mental health needs with physical health concerns, particularly regarding weight gain as a side effect. There was a clear desire for more comprehensive support from healthcare providers in managing these medication‐related weight changes.

These findings align with and extend existing literature in several key areas. Our findings regarding the crucial role of healthcare professional support align with existing literature on patient–provider interactions. Previous research has highlighted tensions between patients valuing general practitioners' roles while simultaneously experiencing insufficient information about antidepressants effects (Cartwright et al. 2016). Similarly, studies have identified significant gaps in shared decision‐making processes, particularly regarding antidepressants side‐effect discussions (Coe et al. 2024), but also within discussions around psychotropic medication (Bui et al. 2022). For instance, Bui et al. (2022) found that patients often received little information about their medication, with inadequate support for managing side effects and minimal involvement in decision‐making. These findings should be considered alongside existing literature on weight stigma in healthcare. A recent multinational study of 13 996 adults found that weight stigma from healthcare providers is highly prevalent, with internalised weight bias linked to healthcare avoidance (Puhl et al. 2021). This presents a concerning parallel: while patients need increased support for managing antidepressant‐related weight changes, the prevalence of weight stigma may create additional barriers to seeking such support.

The complex challenges in managing antidepressant‐related weight gain identified in this study complement previous findings in the literature. Despite using common strategies for weight management (i.e., adjusting their diet and regular exercise), our participants reported significant psychological barriers, including impacts on self‐esteem, negative body image and past difficulties with weight management. Similar challenges have been reported in the literature, with interventions designed to address weight gain associated with psychotropic medication (including antidepressants) showing mixed success (Mccloughen and Foster 2011). Research suggests that previous unsuccessful weight loss attempts may impact future weight management efforts, potentially due to increased frustration and decreased motivation. Research on psychotropic‐related weight gain (including antidepressants) highlights its distressing impact on quality of life and its influence on treatment adherence (Mccloughen and Foster 2011). Their review suggests that existing weight management interventions often have modest results and may not fully address the needs of individuals affected by medication‐related weight gain. Similarly, our findings indicate that adults taking antidepressants could benefit from tailored weight management support that considers both their medication context and personal experiences with weight management.

The mental‐physical health trade‐off described by our participants mirrors issues highlighted in previous research, where individuals faced challenges in establishing and maintaining a therapeutic relationship with healthcare providers (Bui et al. 2022). While our study focused on individuals taking antidepressants, some participants similarly described the struggle of balancing their mental wellbeing with physical health concerns, particularly when medication education and support were lacking. This suggests that regardless of the severity of mental health conditions, individuals taking psychiatric medications may face comparable challenges in weighing the benefits of treatment against side effects. Our findings contribute to understanding these experiences specifically in the context of antidepressant use, suggesting a need for support strategies that acknowledge and address this complex balance.

While previous research identified gender‐specific barriers in weight management (Elliott et al. 2020), our study found no notable differences between men and women's experiences. However, regarding the duration of antidepressant use, our findings suggest potential differences: nearly all long‐term users reported weight gain compared to half of short‐term users. This observation aligns with the literature highlighting the importance of providing early information about long‐term effects and their management (McDonald et al. 2024; Bowers et al. 2020). The lack of such information was a significant concern for our participants, suggesting that early discussions about long‐term side effects and management strategies could be crucial for informed decision‐making about antidepressant use.

### 5.2 Strengths and Limitations

This study benefits from an in‐depth qualitative dataset that allowed for comparisons across gender and long‐term antidepressant use, providing insights into weight management and mental health support. However, several limitations should be noted. First, weight changes and medication use were self‐reported, which may introduce bias, as was the lack of diagnostic data for participants. The absence of detailed diagnostic information on depression subtypes limits the ability to explore how these factors may influence participants' experiences with specific diagnoses. The qualitative nature of the study also means our findings are inherently interpretative and should be applied cautiously. Participants were recruited through convenience sampling on social media, likely attracting individuals comfortable discussing sensitive topics, which may not represent the population of adults prescribed antidepressants. Another limitation of this study is the lack of ethnic diversity among participants, which may limit the generalisability of the findings to more diverse populations. Lastly, while several participants expressed interest in the study, a portion ended up not enrolling in the interview process, which could further limit representation.

### 5.3 Recommendations for Practice

The findings from this study highlight the critical need for enhanced training for healthcare providers on managing weight‐related side effects of antidepressants, emphasising a person‐centred approach. Relatedly, an ongoing evaluation of a virtual patient training tool (Nichol et al. 2024) could offer valuable insights into how such training resources can support healthcare providers in these weight‐management conversations. Implementing an integrated care model that concurrently addresses both mental and physical health concerns could foster collaboration between mental health professionals and weight management specialists. Furthermore, tailored weight management programmes specifically designed for individuals on antidepressants should be enhanced and implemented to effectively address their unique challenges. A pragmatic example could involve using the Making Every Contact Count (MECC) approach, where healthcare professionals integrate brief, targeted weight management conversations into existing mental health consultations (Kemp et al. 2024). Comprehensive educational materials (e.g., low‐cost digital resources) about potential weight‐related side effects and effective management strategies are essential for patients, drawing from existing interventions developed in the context of discontinuation (Bowers et al. 2020). Regular monitoring and follow‐up appointments are also necessary to track weight changes and adapt treatment plans accordingly. Collectively, these recommendations aim to improve the overall healthcare experience for antidepressant users and mitigate the negative impact of weight‐related concerns on their mental health treatment.

## 6 Conclusions

This study highlights three key areas requiring attention in supporting individuals managing weight while taking antidepressants. First, there is a clear need for improved healthcare professional support, particularly regarding proactive discussions about weight management and side effects. Second, weight management strategies need to be tailored to account for the specific challenges faced by individuals taking antidepressants, considering both their medication context and previous experiences. Third, healthcare providers need to acknowledge and address the complex balance between mental and physical health outcomes when prescribing antidepressants. The findings suggest that early intervention, comprehensive support, and regular monitoring could significantly improve experiences of weight management in this population. Future research should focus on developing and evaluating targeted interventions that address these needs, particularly exploring strategies for early support provision and the role of healthcare professionals in facilitating successful weight management outcomes.

### 6.1 Relevance for Clinical Practice

Healthcare professionals should prioritise proactive and tailored conversations about weight management when prescribing antidepressants, emphasising the importance of person‐centred care. Integrating mental and physical health support through collaborative care models can enhance patient outcomes. This includes offering comprehensive educational materials on managing weight‐related side effects and ensuring regular monitoring and follow‐ups to adapt treatment plans. Early intervention and tailored weight management strategies are critical to addressing the unique challenges faced by patients, ensuring a holistic approach to their mental health treatment.

## Author Contributions

A.R. conceived the study idea and developed the initial study design, including the topic guide. All authors (A.R., N.K., C.W., K.B.) approved the final manuscript. C.W. and K.B. contributed to refining the study design, prepared study materials, conducted data collection and prepared summary reports. A.R. and N.K. analysed the final dataset, co‐wrote the manuscript and its subsequent revisions, with all authors providing feedback on data analysis, interpretation and manuscript drafts. All co‐authors have reviewed and approved the final version of the manuscript submitted for publication. A.R. served as the guarantor of the work.

## Ethics Statement

Ethical approval for this study was granted by the Faculty of Health Sciences ethics department at Northumbria University (ref: 45339; Watson 2023‐6122‐5523).

## Consent

All participants provided online consent to participate.

## Conflicts of Interest

The authors declare no conflicts of interest.

## Supporting information


Data S1.

## Data Availability

The datasets used and/or analysed during the current study are available from the corresponding author on reasonable request.
