# Supplementary material for: Optimising Weight Management Advice for Adults Prescribed Antidepressants: A Qualitative Interview Study of Experiences
Source: Int J Ment Health Nurs. 2025 May 28;34(3):e70068. doi: 10.1111/inm.70068 (PMC12120215; doi:10.1111/inm.70068)
Supplement: Supplementary file 1 — Data S1. [file INM-34-0-s001.docx]

**Supplement 1: Interview Topic Guide**

[INTRODUCTION]

Thank you for agreeing to take part in this interview.

- Remind of voluntary nature of the interview.
- Can stop the interview at any time
- Data is anonymous and all identifiable information will be changed/redacted during transcription etc.

How long you have been suffering from [diagnosis]? (Probe for: pre and post diagnosis)

What is your understanding about what [diagnosis] is?

What treatment/s have you had/do you know what is available?

[BELIEFS ABOUT MEDICATION AND WEIGHT]

Could you tell me a bit about the medication that you are currently taking please?

How has it been for you? Have you found that it is useful?

Had you been offered any other medication for your mental health? Were they effective?

How did you feel about this?

Are you aware of other interventions or treatments that are available?

Do you feel as though you were given appropriate support and advice at the time of prescription? Was it a difficult decision to make? Do you feel as though you had enough resources to decide?

What do you think the link is, if at all, between weight and [diagnosis and/or medication]?

Probes: Why? Why not?

Did you feel at all able to deal with any potential side effects of said medication?

[EXPERIENCES OF COMMUNICATION ABOUT WEIGHT MANAGEMENT]

How do you feel about communicating with healthcare professionals about weight management?

Probes: Is it easy, comfortable, difficult? Who typically initiates these discussions?

Can you recall a time when a healthcare professional introduced the topic of weight management? What did they say, and how did that make you feel?

Probe: Can you give an example of what was said or done?

Are there times when weight hasn’t been discussed but you would have wanted it to be mentioned?

Probes: What might be barriers to bringing it up?

How do you feel when healthcare professionals discuss weight management with you?

Probes: Comfortable, uncomfortable, at ease, uneasy?

When discussing weight, how do you prefer healthcare professionals to approach the topic?

Probes: Direct, friendly, empathic, sensitive? Preferred language (e.g., "overweight," "BMI")?

How would you sum up your general experience communicating about weight with healthcare professionals?

[TRAINING AND RESOURCE NEEDS FOR HEALTHCARE PROFESSIONALS]

Reflecting on these conversations, is there anything healthcare professionals could have done or said differently?

Do you think healthcare professionals could benefit from additional training to improve communication about weight management?

Probe: Specific skills or approaches that might be beneficial?

How do you feel about the resources, information, and support available to you to help with weight management?

Probe: Are there particular areas you feel could be expanded or improved?

What advice would you offer to healthcare professionals discussing weight management with people starting antidepressants?

[INFLUENCES ON WEIGHT MANAGEMENT MOTIVATION]

What motivates you to manage your weight? Is there anything healthcare professionals could do to support this motivation?

How confident are you in your own ability to manage weight?

Probe: Do people around you discuss or support weight management?

Are there any specific qualities in a healthcare professional (e.g., empathy, communication skills) that make you more likely to listen or feel motivated to change your behaviour?

How might a healthcare professional help you manage weight effectively?

Probes: Setting specific goals, rapport building, showing understanding.

When do you think is the best time for a healthcare professional to discuss weight management?

Probes: Before prescribing certain medications, during a check-up, linked to other conditions like diabetes or hypertension?

[FINAL THOUGHTS AND ADDITIONAL COMMENTS]

Is there anything we haven’t discussed that you feel is important to add?

Do you have any questions for me?

Thank you very much for your time. I’ll send you a debrief sheet after this conversation, which includes resources and support services relevant to our discussion. If you have any follow-up concerns, especially regarding medication, please discuss them with your GP.
